# Psychopharmacology and Biological Studies of Psychosis

**DOI:** 10.3390/brainsci13060854

**Published:** 2023-05-25

**Authors:** Marcin Siwek, Bernadeta Szewczyk, Adrian Andrzej Chrobak

**Affiliations:** 1 Department of Affective Disorders, Jagiellonian University Medical College, Kopernika St. 21a, 31-501 Krakow, Poland; 2 Maj Institute of Pharmacology Polish Academy of Sciences, Smętna St. 12, 31-343 Krakow, Poland; szewczyk@if-pan.krakow.pl; 3 Department of Adult Psychiatry, Jagiellonian University Medical College, Kopernika St. 21a, 31-501 Krakow, Poland; adrian.chrobak@uj.edu.pl

In most cases, psychotic episodes occur in the course of chronic mental illnesses, e.g., schizophrenia, schizoaffective disorder or bipolar disorder. Disorders such as these are associated with relapses, limited functioning, poor quality of life and shorter life expectancy. Despite the more than 70-year history of antipsychotic drug development and use, not to mention the ongoing search for other therapeutic methods, the current therapy of psychoses faces numerous challenges. It is difficult, and in some cases impossible, to achieve both symptomatic and functional remission. The challenges faced include: the unsatisfactory effectiveness of antipsychotics in the treatment of negative or affective symptoms and cognitive dysfunction; the limited effectiveness of relapse prevention treatment; the frequent problem of drug resistance; the difficulties in achieving functional recovery; the side effects and somatic complications associated with the use of psychotropic drug;, the difficulties in developing new, clinically useful methods of treatment; low therapeutic adherence; and somatic comorbidity. These issues contribute to the deterioration of general functioning and cognitive impairment, increase drug intolerance and narrow the choice of therapeutic methods. Therefore, there is a wide-ranging need for a further deep exploration of the biological basis of psychoses and to search for new methods of pharmacological treatment [1,2]. The articles published in this Special Issue highlight the significant advances of recent years and encourage further investigation in this field.

The exploration of links between schizophrenia symptoms and biomarkers of inflammation, oxidative stress and kynurenine pathway dysfunctions may lead to the individualization of treatment and increase its effectiveness. Więdłocha et al. have shown that oxidative stress biomarkers may be associated with the severity of schizophrenia symptoms in positive, negative and cognitive dimensions. The identification of biochemical markers associated with specific symptom clusters may increase the understanding of biochemical profiles in schizophrenia patients [3].

The only antipsychotic drug recommended by the FDA for treatment-resistant schizophrenia is clozapine. Its superior capacity over all other neuroleptics to treat this condition has been consistently proven in multiple studies. However, the efficacy of clozapine monotherapy is far from optimal, as 40% to 70% of patients fail to respond or present only a partial response to an adequate treatment using this drug. One of the common strategies for overcoming this issue involves the combination of clozapine with another antipsychotic drug. Siwek et al. presented the first retrospective chart review of lurasidone augmentation of clozapine treatment effects in a group of treatment-resistant schizophrenia patients. Their work showed that the use of the abovementioned combination is associated with a reduction in positive, depressive and anxiety symptoms, as well as improvements in psychosocial functioning [4].

Learning and memory deficits accompany numerous brain dysfunctions, including schizophrenia and Alzheimer’s disease (AD), and many studies suggest the role of nitric oxide (NO) in these processes. Cieślik et al. investigated the activity of NO releasers and a selective inhibitor of neuronal NO synthase (nNOS) in preventing the occurrence of short-term memory deficits. The results indicate that scopolamine-induced dysfunctions in hippocampal-dependent spatial learning and memory can be alleviated via treatment with NO-related agents [5].

In psychiatric research, there is a pressing need to develop a short-duration, no-cost, digitalized, point-of-care cognitive assessment tool that integrates both subjective and objective measures of cognition. The THINC-it tool is an easy-to-use mobile application (THINC-it) that can be used to screen and measure cognitive functions and which has use in more areas than in the treatment of patients suffering from depression. Szmyd et al. presented a pilot study in order to assess patients’ cognitive functioning with the Polish version of the THINC-it tool and to analyze its association with self-reported quality of life. The results of this research suggest that this application may be used as a cognitive measure in adults with schizophrenia in both clinical and research settings [6].

Miron et al. presented an observational, cross-sectional study investigating concomitant use of benzodiazepines and mood stabilizers in stabilized schizophrenia patients. The authors also evaluated the differences in the use of those drugs between individuals using antipsychotics administered orally or in long-acting injections. The results show that the long-term use of benzodiazepines and mood stabilizers remains high among stabilized schizophrenia patients, regardless of the antipsychotic formulation (oral or long-acting injections). Patients receiving second-generation long-acting injections of antipsychotics seem to be more likely to be stabilized on monotherapy compared to those receiving oral antipsychotics [7].

Psychotic treatment-resistant depression is a complex and challenging manifestation of mood disorders encountered in the clinical setting. This disorder is often underdiagnosed and undertreated. Ketamine appears to have rapid and potent antidepressant effects in clinical studies. Gałuszko-Węgielnik et al. assessed the use of ketamine to treat major depressive disorder with psychotic features as an add-on treatment to the standard of care. The results suggest that abovementioned therapy may be beneficial in this clinical group [8].

Brain-derived neurotrophic factor (BDNF) is a key modulator of neuroplasticity and has an important role in determining the susceptibility to severe psychiatric disorder with a significant neurodevelopmental component such as major psychoses. Manchia et al. aimed to evaluate the relationship between BDNF serum levels and several demographic, clinical, and psychometric measures in 105 patients with schizophrenia and schizoaffective disorder and assessed the moderating effect of genetic variants within the BDNF gene. This study revealed that BDNF serum levels are significantly lower in patients affected by schizophrenia and schizoaffective disorders presenting more severe depressive symptomatology [9].

There have been reports of taste disturbances in the occurrence of mental disorders. Studies have found a possible relationship between deficit symptoms of schizophrenia and the dysgeusia of monosodium glutamate (MSG). Humans describe all foods as tasting sweet, sour, bitter, or metallic. Wroński et al. aimed to verify whether the level of MSG taste perception is related to the severity of deficit symptoms. The authors found a significant negative correlation between mean intensity of taste and the number of deficit symptoms. The study suggest that the symptoms of taste disturbances reported by the patient should be monitored by clinicians and in order to differentiate between the actual deficits in the domain of taste perception and taste hallucinations as a symptom of psychosis [10].

This Special Issue also included four high-quality quality review papers summarizing current knowledge on important topics related to psychopharmacology and biological studies of psychosis such as: the interplay between schizophrenia, oxytocin and estrogens; epigenetic targets in schizophrenia development and therapy; the application of antipsychotic drugs in mood disorders; as well as the pharmacologic properties and use of brexpiprazole in schizophrenia and mood disorders [11,12,13,14].

In summary, these articles reflect important advances in the active area of psychopharmacology research and biological studies of psychosis. We would like to thank all of the authors who submitted their work to this Special Issue and the reviewers for dedicating their time to improving the quality of the published manuscripts.
